# Central Sensitization-Related Symptoms and Influencing Factors on Health-Related Quality of Life among Frail Older Adults in Senior Day Care Centers: A Cross-Sectional Study

**DOI:** 10.3390/healthcare12121201

**Published:** 2024-06-15

**Authors:** Yuki Kikuchi, Hideki Nakano, Teppei Abiko, Akio Goda, Shin Murata

**Affiliations:** 1 Department of Physical Therapy, Faculty of Health Sciences, Kyoto Tachibana University, Kyoto 607-8175, Japan; 2 Graduate School of Health Sciences, Kyoto Tachibana University, Kyoto 607-8175, Japan; 3 Department of Physical Therapy, Faculty of Health and Medical Science, Hokuriku University, Kanazawa 920-1180, Japan

**Keywords:** health-related quality of life, timed up and go test, functional mobility, frail older adults, day care service

## Abstract

The recent increase in the number of frail older adults has led to increased attention being paid to care services in communities such as senior day care centers. Maintaining health-related quality of life (HRQOL) in frail older adults is important for managing long-term care. The purpose of this study was to comprehensively explore the impact of physical, mental, and cognitive factors, particularly central sensitization-related symptoms (CSSs), on the HRQOL among frail older adults in senior day care centers. HRQOL, physical, mental, and cognitive factors, and severity of CSSs were comprehensively measured using validated methods. Correlation and multiple regression analyses were used to examine factors affecting HRQOL among frail older adults in senior day care centers. The results showed that the timed up and go test significantly affected the HRQOL among frail older adults at senior day care centers. Additionally, knee extension muscle strength, number of pain sites, depressive tendencies, and CSS severity showed a significant negative correlation with HRQOL but were not significant influencing factors. This suggests that functional mobility assessments and approaches are important for maintaining and improving the HRQOL in frail older adults at senior day care centers.

## 1. Introduction

Many older adults wish to age in their own homes and communities and live independent daily lives [1]. However, the number of frail older adults is increasing worldwide, thus increasing the demand for long-term care in the community [2,3]. Special attention is paid to day care facilities and other day care and community-based services, as well as to health support for frail older adults who use these services [4]. Decreasing physical, mental, and cognitive factors among older adults can worsen caregiving levels and reduce health-related quality of life (HRQOL) [5,6]. Reduced HRQOL may result in a shorter healthy life expectancy, limitations in daily activities, hospitalization risk, and increased mortality [7]. Therefore, it is important to extend healthy life expectancies by maintaining and improving the HRQOL of older adults.

Recently, the association between HRQOL and central sensitization-related symptoms (CSSs) was reported in patients with musculoskeletal disorders, whose primary symptom is pain [8,9], and in postoperative females with breast cancer [10]. CSSs are a collective concept that refers to symptoms that have central sensitization (CS) as a common pathological basis [11], hyperalgesia [12], somatic symptoms [13], sleep disturbances [14], chronic fatigue [15], and cognitive dysfunction [16,17]. This phenomenon is often present in older adults in general and can affect their HRQOL with or without pain [18]. Regarding community-dwelling older adults, Kikuchi et al. [19] found that pain intensity and CSS severity affected HRQOL. However, this study included community-dwelling older adults who were independent in daily living and did not include community-dwelling older adults who received care.

Previous studies investigating the factors affecting HRQOL among community-dwelling older adults receiving long-term care are limited. Kitamura et al. [20] found that cognitive decline was associated with HRQOL among non-institutionalized older adults who received long-term care insurance. However, the previous study focused on cognitive function, and handgrip strength was the only measure of physical function assessed. Furthermore, pain severity and CSSs, which have been suggested to be significantly related in previous studies on community-dwelling older adults who were independent in daily living, were not included in the index, which made it insufficient for examining factors related to HRQOL, a multidimensional concept.

The purpose of this study was to comprehensively explore the impact of physical, mental, and cognitive factors, particularly CSSs, on HRQOL among frail older adults in senior day care centers. It is hoped that this study will provide a comprehensive approach to preserve and improve HRQOL among community-dwelling frail older adults in senior day care centers.

## 2. Methods and Materials

### 2.1. Study Design

This observational cross-sectional study was conducted in 2021 in senior day care centers for older adults in Otsu, Shiga Prefecture, Japan. Participants were fully informed of the study’s purpose, content, risks, and benefits, as well as their right to privacy and their ability to withdraw consent and decline participation at any point during the study period. The study was conducted in accordance with the 1975 Declaration of Helsinki (revised in 2013). Ethics approval was obtained from the University of Kyoto Tachibana Research Ethics Committee for conducting this study (approval number: 18–26).

### 2.2. Participants

**Figure 1:**
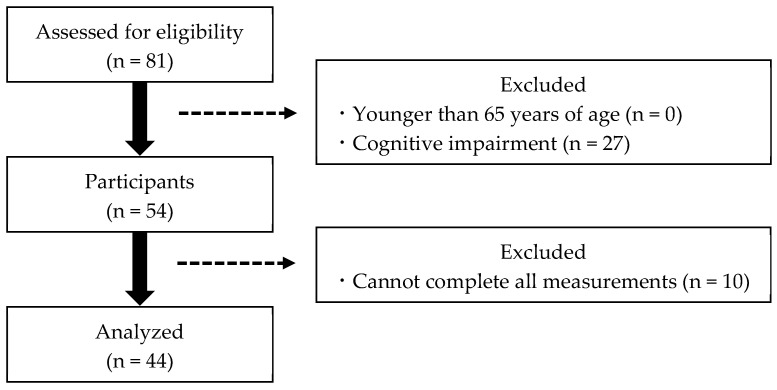
Participant selection flowchart.

This study included 81 community-dwelling frail older adults at senior day care centers who required light care in their daily lives. All the participants received the service according to the Japanese long-term care insurance (LTCI) system [21]. The inclusion criteria were as follows: (1) older adults using the Japanese LTCI system, (2) older adults aged 65 years or older, and (3) the ability to complete all measures. Exclusion criteria were as follows: (1) aged below 65 years, (2) inability to complete all the measured items, and (3) suspected dementia. Suspected dementia was defined as a Mini-Mental State Examination (MMSE) score of less than 24 [22]. Our study was conducted on frail older adults using day care centers and did not exclude them because of illness. Only patients whose vital signs, such as blood pressure and temperature, were checked prior to measurement and those who were allowed to participate in the study were included. We collected data on participants’ sex, age, height, weight, and body mass index. Participants then completed physical, mental, and cognitive assessments to measure their CSS severity. After excluding participants who met the exclusion criteria, 44 participants were included in the final analysis (Figure 1). For confirmation that the sample size was adequate, a post hoc power analysis was performed using G*Power 3.1.9.7. The Results section comments on the findings regarding sufficient power (power ≥ 0.800) [23].

### 2.3. Assessment

We conducted a physical fitness assessment and questionnaire survey to measure HRQOL, physical, mental, and cognitive factors, and CSS severity in community-dwelling frail older adults, using methods similar to those published by Kikuchi et al. [19]. However, for the assessment of lower limb function, a 10 s chair rise test for frail older people (Frail CS-10), designed to be more demographically compatible, was incorporated, unlike previous approaches that utilized a 30 s chair rise test (CS-30). All questionnaires were administered face-to-face.

#### 2.3.1. Health-Related Quality of Life (HRQOL)

To assess HRQOL, we employed the EuroQol 5-Dimensions 5-Levels (EQ-5 D-5L) questionnaire (see Table 1) [24]. This self-administered instrument assesses five dimensions—mobility, self-care, usual activities, pain/discomfort, and anxiety/depression—each on a five-level scale ranging from no problems to extreme problems [25]. Participants’ overall health status is represented through a combination of these scores, with “11111” indicating the absence of any problems and “55555” indicating extreme difficulties across domains [25]. These scores were then converted to utility values or HRQOL scores, with 0 representing death and 1 representing full health status, according to the conversion chart provided by the EuroQol group [26].

#### 2.3.2. Physical Factors

The physical factors assessed were physical function and pain levels. Grip and knee extensor muscle strengths were determined using methodologies similar to those described in previous studies [27]. A digital grip strength meter (T.K.K. 5401; Takei Kiki Kogyo Co., Niigata, Japan) was used to measure grip strength. Participants bent the proximal interphalangeal joints of their fingers at right angles and grasped the digital grip strength meter so that the display side of the meter was visible to the evaluator. In the initial posture, participants stood upright and placed their feet shoulder-width apart while allowing their arms to dangle freely. Participants were instructed to grasp the digital grip strength meter with maximum force, being careful to avoid contact between the device and their body or clothing. Knee extension muscle strength was assessed using a muscle strength measuring table for one leg (T.K.K. 5715; Takei Kiki Kogyo Co., Ltd., Niigata, Japan). Participants were fitted with a tension meter (T.K.K. 5710 (e); Takei Kiki Kogyo Co., Ltd., Niigata, Japan) at their ankles. Participants were instructed to sit on a single-leg strength test table, cross both upper extremities in front of the chest, and extend the knee with maximum force.

Lower limb function was measured using the Frail CS-10, modified from the CS-30 developed by Jones et al. [28,29] for frail older adults by Murata et al. [30]. In the Frail CS-10, the participants were seated on a chair without armrests, with both upper limbs at the knee as the starting limb position. Measurements were taken for 10 s, and the number of completed cycles of sitting, standing, and being seated was recorded. The Frail CS-10 has already been confirmed as reliable and valid for the assessment of lower limb function [30].

Balance capacity was assessed by conducting the one-leg standing test described by Goda et al. [31]. The participants were instructed to keep their arms by their sides while focusing on a preset marker positioned at eye level, two meters away. The assessment was interrupted if any of the following three conditions occurred: first, if the lifted foot encountered the supporting foot or ground; second, if any movement of the supporting foot occurred; and third, once the duration reached 120 s for each trial on either side. The procedure was repeated twice on each side for each participant, and the average of the longest times on each side was recorded as the final measure.

The timed up and go test (TUG) was administered to evaluate functional mobility in older adults following the protocol outlined by Kurosawa et al. [32]. In this study, we measured the duration required for a participant to rise from a seated position, navigate around a marker placed 3m away, and return to the chair. Instructions were given to the participants to complete the gait as quickly as possible to promote uniformity in test performance.

Pain ratings encompassed three aspects: the existence of pain over the past month, intensity of pain experienced, and number of pain sites. The participants were asked about their physical pain experience over the past month with a simple “yes” or “no” response [19]. They were then asked to specify the location(s) of pain from a predefined list, including the head, neck, shoulder, back, hip, knee, and ankle joints. Pain intensity was quantified using a numerical rating scale proven for its reliability and validity [33], with ratings spanning from “0”, indicating no pain, to “10”, representing the most severe pain imaginable.

#### 2.3.3. Mental Factors

Mental factors were assessed using the Geriatric Depression Scale (GDS), a depression scale developed by Yesavage [34] for older adults. A shortened version of GDS-5 [35,36] was used in this study. GDS-5 has been validated as a screening tool for depression in older adults. The scale consists of questions which can be answered by either “yes” or “no”. Importantly, scoring is determined by the nature of the question, with one point assigned for each negative response that indicates a depressive tendency and zero points for positive responses that do not indicate depression, leading to a scoring system in which higher totals indicate more severe depressive symptoms [34].

#### 2.3.4. Cognitive Factors

Cognitive and attentional functions were assessed as components of cognitive factors. The MMSE [37], a validated screening tool for dementia, was used to assess cognitive function. The MMSE includes activities such as writing, constructing sentences, drawing, and offering a comprehensive gauge of the cognitive state. The total MMSE score on the MMSE is 30 points, with scores of 23 or below indicating concerns about possible dementia [22].

Attentional function was evaluated using the Trail Making Test (TMT). TMT Part A (TMT-A) [38,39], recognized for its reliability and validity, was employed to assess participants’ performance. Participants were given a sheet with random numbers ranging from 1 to 25 and instructed to link these numbers with a line in ascending order. The duration for the participant to successfully complete the task (connecting all numbers) was recorded, with longer times indicating poorer attentional functioning.

#### 2.3.5. Central Sensitization-Related Symptoms (CSSs)

CS is characterized by an enhanced response of nociceptive neurons within the central nervous system to stimuli that are typically normal or below the threshold [40]. Increased CS is an essential pathological factor underlying central sensitivity syndrome that results in a spectrum of physical and psychological manifestations [41]. The central sensitization inventory (CSI) serves as the primary assessment tool to quantify CSS severity [42]. It consists of two sections: Part A, which features 25 questionnaires on CSSs, and Part B, which queries the diagnoses of eight specific CSS conditions [43]. In this study, CSS severity was gauged using a short form of CSI known as CSI-9. This short-form version has been validated for its high reliability and efficacy as a diagnostic tool for identifying CSSs in clinical settings. The CSI-9 encompasses nine items of questions, each answered on a scale from 0 (never) to 4 (always) (see Table 2) [43]. This scoring mechanism allows scores to range from 0 to 36, with elevated scores signifying increased CSS severity. In previous studies, a CSI-9 score of over 20 points indicated the presence of significant CSS [44].

### 2.4. Statistical Analysis

We performed a statistical analysis to correlate HRQOL with physical, mental, and cognitive factors and CSS severity in 44 participants. We confirmed the association between HRQOL and sex by calculating the correlation ratios. To further examine the factors influencing HRQOL, a multiple regression analysis was conducted using the forced entry method. HRQOL was included as the dependent variable, while factors that showed significant correlations with HRQOL were included as independent variables. Multicollinearity was considered during multiple regression analysis by calculating the variance inflation factor (VIF) and ensuring that the VIF was <5 [45]. Statistical analysis was performed using IBM SPSS Statistics for Windows, Version 29.0 (Armonk, NY, USA), with a significance level of 5%. 

## 3. Results

Basic attributes and the results of each measurement are shown in Table 3. The participants’ mean age is 82.1 (5.3) years, height is 153.0 (9.6) cm, weight is 55.6 (11.6) kg, and body mass index is 23.7 (4.0) kg/m^2^. The mean (standard deviation) HRQOL score for the main outcome is 0.652 (0.157). The mean (standard deviation) CSI-9 score is 10.3 (6.7). Five participants (11.4%) exceeded the severe CSS cutoff of 20 points.

The correlation analysis revealed that the item that showed a significant positive correlation with HRQOL was knee extension muscle strength (r = 0.357, *p* = 0.017). Further items that showed significant negative correlations with HRQOL were TUG (r = −0.448, *p* = 0.002), GDS-5 score (r = −0.358, *p* = 0.017), number of pain sites (r = −0.462, *p* = 0.002), and CSI-9 score (r = −0.546, *p* < 0.001) (Table 4 and Table 5).

In addition, a multiple regression analysis was performed using HRQOL as the dependent variable and factors that demonstrated significant correlations with HRQOL as the independent variable. Our findings highlighted TUG (β = −0.352, *p* = 0.007) as a factor significantly influencing HRQOL of frail older adults (refer to Table 6). The VIF values of the independent variables (knee extension, TUG, number of pain sites, GDS-5 score, and CSI-9 score) ranged from 1.046 to 1.832, suggesting no issues with multicollinearity. The calculated power from the post hoc power analysis was 0.983, confirming sufficient power.

## 4. Discussion

This study investigated the effects of physical, mental, and cognitive factors and CSS severity on HRQOL in community-dwelling frail older adults at senior day care centers. The results showed that TUG was selected as the factor significantly influencing HRQOL. TUG is a performance test that evaluates dynamic balance and functional mobility in frail older adults; it involves standing from a sitting position, walking 3 m, turning, returning 3 m, turning, and sitting back down [46]. The results obtained with the TUG predict impairments in activities of daily living and instrumental activities of daily living [47] and have shown a strong association with HRQOL [48]. Olivares et al. [5] investigated the association between physical ability and HRQOL in older adults who are independent in daily living and reported that TUG demonstrated the highest odds ratio for HRQOL. Additionally, Davis et al. [49] found TUG to be a predictor of changes in HRQOL over time. In the present study, the TUG was found to be significantly correlated with HRQOL, and it was a major influencing factor. These findings suggest the importance of functional mobility assessment and approaches to maintain and improve HRQOL in frail older adults at senior day care centers. Furthermore, they suggest that implementing approaches to improve lower limb function, such as exercise therapy, may be beneficial.

In a previous study by Kikuchi et al. [19], employing similar measurements and statistical analysis to the present study, pain intensity and CSS severity were found to influence HRQOL in community-dwelling older adults who are independent in daily living. However, unlike previous similar studies involving community-dwelling older adults, pain and CSS severity were not found to influence HRQOL in our study. This disparity could be attributed to various factors. Specifically, the negative impact of pain on quality of life might be diminished in older age, as pain is considered more “normative” in older adults [50]. Furthermore, older adults and frail older adults might underestimate pain due to the presence of more severe health problems other than pain [51]. Considering that our study participants were older, with a mean age of 82.1 years, and disabled to the extent that they require light care, the presence of pain and CSS might have been perceived as “normative” and masked by the presence of more severe health problems, thereby not influencing HRQOL. Based on this, we considered that TUG, rather than pain and CSS severity, was a major influencing factor on HRQOL among frail older adults at senior day care centers.

Knee extension muscle strength, GDS-5, and the number of pain sites were significantly correlated with HRQOL among frail older adults at senior day care centers but were not significant influencing factors. These results suggest that muscle strength, depression, and pain problems are not the main factors influencing HRQOL among frail older adults at senior day care centers, underscoring the importance of functional mobility. Additionally, CSS severity was also significantly correlated with HRQOL in frail older adults but did not have a significant influence on HRQOL. Haruyama et al. [52] reported that 4.2% of community-dwelling older adults in Japan had severe CSSs. Kikuchi et al. [19] reported that 2.4% of community-dwelling older adults who were independent in daily living had severe CSSs. In the present study, 11.4% of the older adults had severe CSSs, which was higher than that in previous studies. Our results suggest that although a higher proportion of community-dwelling frail older adults at senior day care centers have severe CSSs than community-dwelling older adults who are independent in daily living, CSS severity is not a factor that significantly affects HRQOL. Tansuğ et al. [53] reported a stronger association between HRQOL and pain in frail institutionalized older adults than in community-dwelling older adults. The absence of an effect of pain on HRQOL in this study suggests that the association between HRQOL and pain may be less relevant in frail older adults living in the community but requiring light care in daily living.

However, because this was a cross-sectional study, it was not possible to determine a causal relationship between functional mobility and HRQOL among frail older adults in senior day care centers. Further longitudinal studies are needed to clarify the causal relationship between functional mobility and HRQOL and devise effective approaches for frail older adults at senior day care centers. Additionally, due to the wide range of comorbidities among the participants in this study, future studies should employ a larger sample size and investigate the impact of comorbidities on HRQOL. Future research should also investigate the roles of social and family support [54], sleep quality [55], and comorbidities [56], which have been previously reported to be associated with HRQOL, and comprehensively investigate the impact of these factors on HRQOL in frail older adults at senior day care centers.

## 5. Conclusions

Our results revealed that functional mobility influences HRQOL in community-dwelling frail older adults at senior day care centers, suggesting the importance of assessing and approaching functional mobility. However, in this study, the severity of pain and CSSs were not influencing factors of HRQOL, suggesting that the factors influencing HRQOL among community-dwelling older adults may exhibit different trends depending on their level of independence in daily living. If future research clarifies the causal relationship between HRQOL and functional mobility, it is expected to lead to more effective interventions and improve HRQOL among community-dwelling frail older adults at senior day care centers.

## Figures and Tables

**Table 1:** EuroQol 5-Dimensions 5-Levels.

**MOBILITY**	
I have no problems in walking	1
I have slight problems in walking	2
I have moderate problems in walking	3
I have severe problems in walking	4
I am unable to walk	5
**SELF-CARE**	
I have no problems with washing or dressing myself	1
I have slight problems with washing or dressing myself	2
I have moderate problems with washing or dressing myself	3
I have severe problems with washing or dressing myself	4
I am unable to wash or dress myself	5
**USUAL ACTIVITIES** (e.g., work, study, housework, family, or leisure activities)	
I have no problems doing my usual activities	1
I have slight problems doing my usual activities	2
I have moderate problems doing my usual activities	3
I have severe problems doing my usual activities	4
I am unable to do my usual activities	5
**PAIN/DISCOMFORT**	
I have no pain or discomfort	1
I have slight pain or discomfort	2
I have moderate pain or discomfort	3
I have severe pain or discomfort	4
I have extreme pain or discomfort	5
**ANXIETY/DEPRESSION**	
I am not anxious or depressed	1
I am slightly anxious or depressed	2
I am moderately anxious or depressed	3
I am severely anxious or depressed	4
I am extremely anxious or depressed	5

**Table 2:** Short form of the central sensitization inventory.

Central Sensitization Inventory-9	
1.	Unrefreshed in the morning
2.	Muscles stiff/achy
3.	Pain all over body
4.	Headaches
5.	Do not sleep well
6.	Difficulty concentrating
7.	Stress makes symptoms worse
8.	Tension in the neck and shoulders
9.	Poor memory

0 = Never, 1 = Rarely, 2 = Sometimes, 3 = Often, 4 = Always.

**Table 3:** Characteristics of participants.

					n = 44
			Mean	±	SD
Age	years		82.1	±	5.3
Sex	n (%)	Male/Female	16 (36.4)/28 (63.6)		
Height	cm		153.0	±	9.6
Weight	kg		55.6	±	11.6
BMI			23.7	±	4.0
HRQOL score			0.652	±	0.157
Handgrip	kgf		20.9	±	6.5
Knee extension	kgf		21.0	±	7.3
Frail CS-10	repetitions		4.8	±	1.6
One-leg standing	seconds		8.7	±	14.7
TUG	seconds		10.3	±	3.4
Pain	n (%)		34 (77.3)		
Pain intensity	points		4.4	±	3.0
Number of pain sites	n (%)	0	10 (22.7)		
		1	13 (29.5)		
		2	15 (34.1)		
		3	4 (9.1)		
		4	-		
		5	1 (2.3)		
		6	-		
		7	1 (2.3)		
GDS-5	points		1.6	±	1.4
MMSE	points		27.7	±	2.1
TMT	seconds		162.1	±	53.1
CSI-9	points		10.3	±	6.7
	n (%)	≥20 points	5 (11.4)		

Notes: SD: standard deviation; BMI: body mass index; HRQOL score: health-related quality of life score; Frail CS-10: 10 s chair stand test for frail elderly; TUG: timed up and go test; GDS-5: Geriatric Depression Scale-5; MMSE: Mini-Mental State Examination; TMT: Trail Making Test; CSI-9: central sensitization inventory-9.

**Table 4:** Correlations of the HRQOL score with basic attributes.

					n = 44
	Age	Sex	Height	Weight	BMI
HRQOL Score	−0.141	0.021 ^a^	0.198	0.132	0.066

Notes: HRQOL, health-related quality of life; BMI: body mass index. a: Correlation ratio.

**Table 5:** Correlations of the HRQOL score with physical, mental, and cognitive factors and CSSs.

						n = 44
	**Handgrip**	**Knee Extension**	**Frail CS-10**	**One-Leg Standing**	**TUG**	
HRQOL score	0.113	0.357 *	0.141	−0.001	−0.448 **	
	**Pain Intensity**	**Number of Pain Sites**	**GDS-5**	**MMSE**	**TMT-A**	**CSI-9**
HRQOL score	−0.259	−0.462 **	−0.358 *	0.027	−0.279	−0.546 **

Notes: HRQOL, health-related quality of life; Frail CS-10, 10 s chair stand test for the frail elderly; TUG, timed up and go test; GDS-5, Geriatric Depression Scale-5, MMSE, Mini-Mental State Examination; TMT, Trail Making Test; CSI-9, central sensitization inventory-9. * *p* < 0.05, ** *p* < 0.01.

**Table 6:** Multiple regression analysis results.

						n = 44
Dependent Variables	HRQOL Score					
		β	95% CI		*p*-Value	VIF
			Lower	Upper		
Independent variables	Knee extension	0.095	−0.004	0.008	0.472	1.164
	TUG	−0.352	−0.028	−0.005	0.007	1.046
	Number of pain sites	−0.267	−0.067	0.006	0.100	1.712
	GDS-5	−0.246	−0.061	0.005	0.089	1.358
	CSI-9	−0.215	−0.013	0.003	0.197	1.832
	R^2^	0.443				
	Adjusted R^2^	0.369				

Notes: 95% CI, 95% confidence interval; VIF: variance inflation factor; HRQOL score, health-related quality of life score; TUG: timed up and go test; GDS-5: Geriatric Depression Scale-5; CSI-9: central sensitization inventory-9.

## Data Availability

The data presented in this study are available upon request from the corresponding author. The data are not publicly available since they contain information that may infringe on the privacy of the study participants.
